# Flexible Conductive Anodes Based on 3D Hierarchical Sn/NS-CNFs@rGO Network for Sodium-Ion Batteries

**DOI:** 10.1007/s40820-019-0294-9

**Published:** 2019-08-01

**Authors:** Linqu Luo, Jianjun Song, Longfei Song, Hongchao Zhang, Yicheng Bi, Lei Liu, Longwei Yin, Fengyun Wang, Guoxiu Wang

**Affiliations:** 1 College of Physics and State Key Laboratory of Bio-Fibers and Eco-Textiles, Qingdao University, Qingdao, 266071 People’s Republic of China; 2 College of Electromechanical Engineering, Qingdao University of Science and Technology, No. 99 Songling Road, Qingdao, 260061 Shandong People’s Republic of China; 3 School of Materials Science and Engineering, Shandong University of Science and Technology, Qingdao, 266590 People’s Republic of China; 4 School of Materials Science and Engineering, Shandong University, Jinan, 250061 People’s Republic of China; 5 Centre for Clean Energy Technology, University of Technology Sydney, Broadway, Sydney, NSW 2007 Australia

**Keywords:** Flexible electrodes, N,S co-doped carbon nanofibers, Reduced graphene oxide, Sn quantum dots, Sodium-ion batteries

## Abstract

Metallic Sn has provoked tremendous progress as an anode material for sodium-ion batteries (SIBs). However, Sn anodes suffer from a dramatic capacity fading, owing to pulverization induced by drastic volume expansion during cycling. Herein, a flexible three-dimensional (3D) hierarchical conductive network electrode is designed by constructing Sn quantum dots (QDs) encapsulated in one-dimensional N,S co-doped carbon nanofibers (NS-CNFs) sheathed within two-dimensional (2D) reduced graphene oxide (rGO) scrolls. In this ingenious strategy, 1D NS-CNFs are regarded as building blocks to prevent the aggregation and pulverization of Sn QDs during sodiation/desodiation, 2D rGO acts as electrical roads and “bridges” among NS-CNFs to improve the conductivity of the electrode and enlarge the contact area with electrolyte. Because of the unique structural merits, the flexible 3D hierarchical conductive network was directly used as binder- and current collector-free anode for SIBs, exhibiting ultra-long cycling life (373 mAh g^−1^ after 5000 cycles at 1 A g^−1^), and excellent high-rate capability (189 mAh g^−1^ at 10 A g^−1^). This work provides a facile and efficient engineering method to construct 3D hierarchical conductive electrodes for other flexible energy storage devices.

**Electronic supplementary material:**

The online version of this article (10.1007/s40820-019-0294-9) contains supplementary material, which is available to authorized users.

## Highlights

- 3D hierarchical conductive Sn quantum dots encapsulated in N,S co-doped carbon nanofibers sheathed within rGO scrolls (Sn/NS-CNFs@rGO) were prepared through an electrospinning process.
- Flexible Sn/NS-CNFs@rGO electrode exhibits superior long-term cycling stability and high-rate capability in sodium-ion batteries.

**Electronic supplementary material:**

The online version of this article (10.1007/s40820-019-0294-9) contains supplementary material, which is available to authorized users.

## Graphical abstract

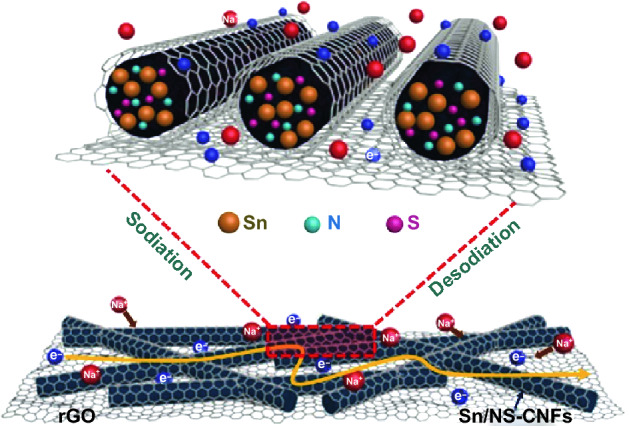

## Introduction

Rechargeable sodium-ion batteries (SIBs) have attracted increasing attention for large-scale energy storage in renewable energy and smart grid applications, owing to their favorable energy density, natural abundance, and low cost [1–5]. However, the larger size of Na^+^ (1.02 Å in radius) vs. Li^+^ (0.76 Å in radius) leads to sluggish diffusion kinetics as well as large volume changes during sodiation/desodiation process, which results in poor cycling stability and inferior rate capability [6–9]. Therefore, developing suitable electrode materials with high theoretical capacity, outstanding cyclic stability, and inexpensive elements is the key for achieving high-performance SIBs [10–14].

Metallic Sn is considered to be a promising anode material owing to its high theoretical capacity (847 mAh g^−1^ with Na_15_Sn_4_), environmental friendliness, and low cost [15, 16]. However, the drastic volume expansion (~ 520%) from Sn to Na_15_Sn_4_ leads to continuous pulverization, resulting in a loss of electrical connectivity to the current collector and a dramatic capacity fading after the initial cycle [17]. To date, many efforts have been devoted to overcome this issue [18, 19]. It demonstrated that nanonization of Sn (especially ~ 5 nm size scale) is an effective strategy to mitigate the mechanical stresses associated with sodiation/desodiation process, thus inhibiting fracture and decrepitation. Moreover, nano-Sn can shorten the Na^+^ transfer lengths, maintaining excellent rate capability [20, 21]. Nevertheless, the improvement achieved by simply reducing the particle size is still unsatisfactory due to a tendency toward aggregation of Sn nanoparticles, which results in poor cycling stability.

Combination with carbonaceous materials is a widely adopted approach to circumvent the pulverization and aggregation issues of Sn nanoparticles during cycling process [21, 22], which can serve as a buffer matrix to alleviate the enormous volume changes of Na–Sn alloying/dealloying and prevent Sn nanoparticle aggregation. The carbonaceous materials also improve the conductivity of electrode and provide additional sodium storage [23]. In recent years, several effective attempts have been made to integrate nano-Sn with one-dimensional (1D) carbon nanofibers (CNFs), attractive for their uniform structure, oriented transport channels for electron and ion, and strong tolerance to mechanical stress [24–26]. 1D CNFs have demonstrated ideal construction units to build multi-functional and multi-dimensional electrodes for advanced energy storage devices [27]. In addition, 2D reduced graphene oxide (rGO) has been extensively explored to enhance the electrochemical performance of Sn-based anode materials in SIBs, owing to its large specific surface, excellent electrical conductivity, and superior mechanical flexibility. It can contribute a conductive matrix and flexible support structure, enhancing electron transport and maintaining electrode integrity [28–32]. Nevertheless, there are still limited reports on the design and fabrication of a nanostructured composite, which combines the advantages of 1D CNFs and 2D rGO.

Recently, N and S heteroatoms have also drawn increasing interest as dopants in carbon-based materials for SIBs. N doping can tune the electron donor/acceptor properties of carbon and also enhance the sodium storage property by creating defects, providing more channels and active sites for Na^+^ absorption [33, 34]. S doping can change the electronic and metallic properties of carbon, and the C–S–C bonds can offer additional electron transfer routes for increasing the electronic conductivity, which indirectly facilitates the enhancement of the sodium storage capability [35].

Inspired by the abovementioned factors, it is highly desirable to combine these strategies into one-electrode design, which could fully exploit the individual and synergistic advantages. Herein, we report an ingenious strategy to fabricate Sn quantum dots (QDs) finely encapsulated in N and S co-doped carbon nanofibers (NS-CNFs) sheathed within rGO scrolls (denoted as Sn/NS-CNFs@rGO) as anodes for SIBs. Such a unique 3D nanoarchitecture ensures a short ion transport/diffusion path, high electronic conductivity, and more electrochemically active sites. Intriguingly, the as-prepared Sn/NS-CNFs@rGO nanoarchitecture, which is woven into a 3D conductive flexible membrane, can be directly employed as binder- and current collector-free electrodes, thus increasing the energy density, lowering the cost, and attracting great interests for next-generation flexible energy storage devices [36]. As a result, based on the optimized structure and synergistic effects between the individual components, these newly designed flexible Sn/NS-CNFs@rGO electrodes displayed outstanding electrochemical performance, including high reversible capacity, excellent rate capability, and ultra-long cyclic stability.

## Experimental Section

### Materials

Thiourea (≥  99.0%), tin chloride dihydrate (SnCl_2_·2H_2_O, 98%), *N*, *N*-dimethyl formamide (DMF, 99.9%), and polyacrylonitrile (PAN, *M*_w_ = 150,000) were purchased from Aladdin. All the chemicals were directly used without further purification.

### Synthesis of Sn/N-CNFs and Sn/NS-CNFs

The Sn/N-CNFs were fabricated through a conventional electrospinning process. 0.7 g of PAN was firstly dissolved in 5 g DMF under vigorous stirring for 6 h at ambient temperature, and then 0.3 g SnCl_2_·2H_2_O was subsequently added. The mixed solution was continuously magnetically stirred at 60 °C for 12 h and served as the precursor solution. The as-prepared solution was loaded into a 5-mL plastic syringe, and a grounded aluminum foil was used to collect the fibers. A high direct current voltage of 15 kV was applied at a distance of 15 cm between the collector plate and needle. The as-spun fibers were firstly dried for about 5 h at 60 °C, and then were pre-stabilized at 250 °C with a heating rate of 2 °C min^−1^ in air for 2 h. Finally, the products were further carbonized at 800 °C for 2 h with a heating rate of 1 °C min^−1^ in Ar. During the calcination, the PAN was transformed to N-doped CNFs (N-CNFs), and SnCl_2_ was reduced to Sn. Eventually, Sn/N-CNFs interconnected into a 3D membrane with the thickness of 109 μm were obtained (Fig. S1). Particularly, the electrode thickness and active material loading of Sn/N-CNFs membrane can be easily controlled by adjusting the electrospinning time (Fig. S2).

The preparation processes of Sn/NS-CNFs are the same as that of Sn/N-CNFs except for the addition of 0.08 g thiourea into precursor solution, which served as the sulfur source and provided additional nitrogen.

### Synthesis of Sn/N-CNFs@rGO and Sn/NS-CNFs@rGO

The flexible free-standing Sn/N-CNFs@rGO and Sn/NS-CNFs@rGO electrodes were easily fabricated by an electrospinning process followed by vacuum filtration. Firstly, GO was fabricated by a modified Hummers method. The concentration of the GO suspension used was 0.5 mg mL^−1^. 20 mg of GO was ultrasonically dispersed in 40 mL distilled water for 3 h to obtain the GO solution. Secondly, the as-spun Sn/N-CNFs and Sn/NS-CNFs precursor nanofibers were employed as a template and filtered 2 mL GO solution through a filter membrane. Subsequently, the obtained filter membrane was dried in the oven at 60 °C overnight, then through the same annealing process as Sn/N-CNFs and Sn/NS-CNFs. Finally, the Sn/N-CNFs@rGO and Sn/NS-CNFs@rGO composites were obtained.

### Materials Characterization

Phase purity and crystal structure of samples were performed by X-ray diffraction (XRD) using Rigaku D/max-rB with Cu Kα radiation (*λ* = 0.15406 nm). The morphologies of the synthesized products were investigated using field-emission scanning electron microscopy (FESEM, Zeiss Supra 55VP) and transmission electron microscopy (TEM) (JEOL JEM 2100F). The elemental distribution was performed by energy-dispersive X-ray spectroscopy (EDS, Oxford Instrument and EDAX Inc). Nitrogen adsorption–desorption isotherms were measured with a Quantachrome Quadrasorb EVO sorption analyzer at 77 K. The samples were degassed in a vacuum at 300 °C for 6 h before the measurements. The specific surface areas were calculated by the Brunauere–Emmette–Teller (BET) method, and pore size distributions were derived from the adsorption branches of the isotherms using the Barrette–Joynere–Halenda (BJH) method. X-ray photoelectron spectroscopy (XPS) measurement was performed on ESCALAB 250 equipment. Thermogravimetric analyses and Raman spectrometry were conducted on Shimadzu DTG-60 and Thermo Scientific DXR2.

### Electrochemical Investigations

LIR2032-type coin cells were assembled in an argon-filled glovebox to investigate the electrochemical performance. The counter and reference electrode were pure sodium metal foil, and 1 M sodium perchlorate (NaClO_4_) dissolved in the mixture of ethylene carbonate (EC) and dimethyl carbonate (DMC) (1:1 in volume) was used as electrolyte. The samples were cut into small disks and directly used as electrodes for SIBs, without mechanical milling or slurry coating processes. Neither a metal current collector nor additives (binder or conductive carbon) were required. The average mass loading of whole electrodes was 0.43 (Sn/N-CNFs and Sn/NS-CNFs) and 0.47 mg cm^−2^ (Sn/N-CNFs@rGO and Sn/NS-CNFs@rGO), respectively, based on the values of 12 samples. The galvanostatic discharge/charge cycling was tested on a LAND battery testing system over a potential range of 0.01–2.0 V versus (Na/Na^+^), the capacity was calculated based on the weight of the whole composite. Cyclic voltammetry (CV) tests were carried out on a CHI 660E electrochemical workstation between 0.01 and 2.0 V. Electrochemical impedance spectra (EIS) were measured in the frequency range of 100 kHz to 10 mHz with an amplitude of 5 mV.

## Results and Discussion

**Fig. 1:**
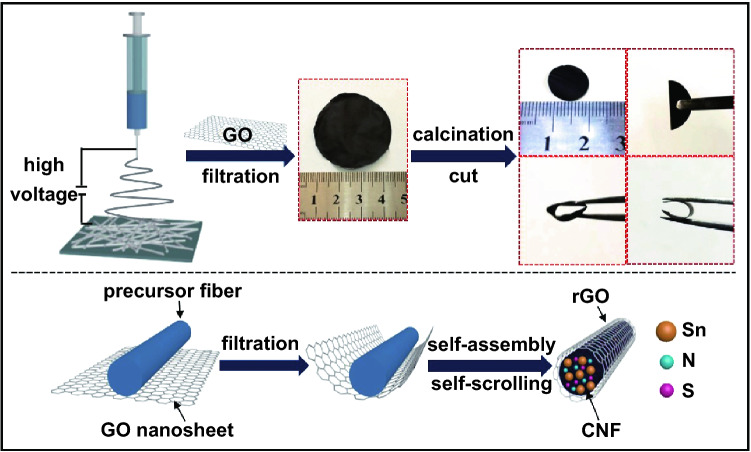
Schematic illustration of the formation process of Sn/NS-CNFs@rGO flexible electrodes

Figure 1 displays the schematic illustration of the fabrication process for Sn/NS-CNFs@rGO flexible electrodes. At first, the precursor solution of SnCl_2_, thiourea, and polyacrylonitrile (PAN) were transformed into large-scale nanofiber membranes by a facile electrospinning technique (Fig. S3a). The membrane consists of ultra-long and continuous precursor nanofibers with uniform diameters about 200–300 nm (Fig. S3b, c). Subsequently, GO sheets were adhered to the surface of as-spun nanofibers through vacuum filtration. After that, a calcination treatment was applied to induce the reduction and self-scrolling of GO sheets bending around the nanofibers, resulting from the recovery of the *π*-conjugated system from GO [27, 37], which leading to the formation of finely defined coaxial core-sheath nanofibers, each units composed of a Sn/NS-CNFs core and a rGO sheath. Finally, the obtained 3D conductive Sn/NS-CNFs@rGO network was cut into self-supporting electrodes with excellent flexibility (even with small-radii 180° bends), which can be directly assembled into SIBs without conductive agent, binder or metal current collectors. More details about experiment can be found in the Experimental Section.

**Fig. 2:**
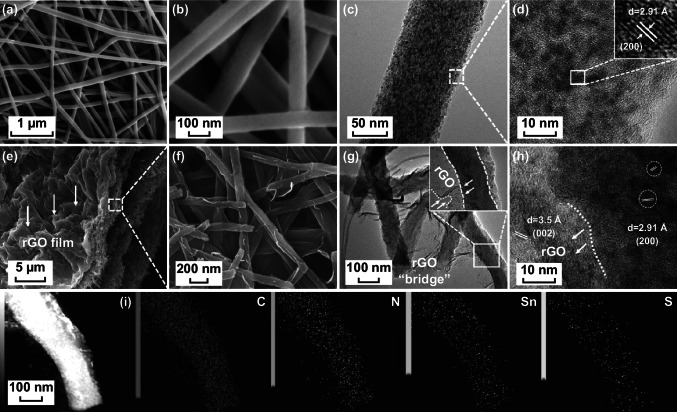
Morphology and microstructure characterizations of Sn/N-CNFs and Sn/NS-CNFs@rGO. **a**, **b** SEM images of Sn/N-CNF; **c** TEM and **d** HRTEM images of Sn/N-CNFs. **e** Low-magnification and **f** high-magnification SEM images of Sn/NS-CNFs@rGO. **g** TEM and **h** HRTEM images of Sn/NS-CNFs@rGO. **i** Dark-field image of the Sn/NS-CNFs@rGO and the corresponding EDS elemental mapping of C, N, Sn, and S, illustrating homogeneous distribution of C, N, Sn, and S atoms along the nanofiber body

The morphology and microstructure of the samples were observed by SEM and TEM. The SEM images (Fig. 2a, b) of Sn/N-CNFs show that the long nanofibers with a uniform diameter (~ 100 nm) are interconnected into a 3D network structure, and the decreased diameter from precursor nanofibers is mainly because of the loss of PAN during annealing process. In addition, the Sn/NS-CNFs exhibit similar morphology to that of Sn/N-CNFs (Fig. S4). The 1D structure is expected to reduce the diffusion paths for Na^+^ and ensure efficient electron transfer during cycling [24, 38]. TEM image (Fig. 2c) shows that Sn QDs (black dots) are homogeneously encapsulated in N-CNFs, and no Sn QDs are found on the surface. The HRTEM image (Fig. 2d) reveals parallel fringes with a lattice spacing of 2.91 Å can be ascribed to the (200) plane of crystalline Sn, and the Sn QDs with a size below 5 nm are uniformly embedded in N-CNFs. As a result, the carbon matrix can not only prevent the aggregation of Sn QDs, but also avoids the exfoliation of Sn QDs during sodiation/desodiation. Figure S5a exhibits the N_2_ adsorption–desorption isotherm of Sn/N-CNFs, manifesting a typical IV-type behavior with a distinct hysteresis loop at relative pressures P/P_0_ ranging from 0.45 to 0.95, implying that Sn/N-CNFs contain a large amount of mesopores [39]. Accordingly, Sn/N-CNFs possess a large Brunauere–Emmette–Teller (BET) specific surface area of 413 m^2^ g^−1^. Figure S5b further confirms that the pore size distribution of Sn/N-CNFs show mesoporous features and the pore sizes in the range of 4–12 nm. The porous structure provides versatile transport pathways for electrolyte ions and enables fast Na^+^ transport.

To demonstrate the delicate microstructure of Sn/NS-CNFs@rGO, SEM images at low and high magnification are displayed in Fig. 2e , f, respectively. The cross-sectional image of Sn/NS-CNFs@rGO (Fig. 2e) displays an interconnected hierarchical 3D structure. The 2D rGO film was constructed by filtration of the initially isolated GO nanosheets with a subsequent calcination treatment. These can act as a current collector and benefit the charge transfer. Further compression of 2D rGO films causes a collapse, and they interconnect with Sn/NS-CNFs into a 3D structure. It should be noted that the 1D nanostructure is essential for the successful construction of the intact 3D structure, and the wrinkles in rGO films/membranes are beneficial for tolerating applied pressures. Figure 2f clearly displays the final rGO sheets sheathing the NS-CNFs surface, and the NS-CNFs are cross-linked with each other due to their connections with the rGO. Taken together, the Sn/N-CNFs@rGO also presents similar morphology with that of Sn/NS-CNFs@rGO (Fig. S6).

TEM and HRTEM images (Figs. 2g–h and S7) were used to reveal the detailed nanostructures of Sn/NS-CNFs@rGO. Figures 2g and S7 present the Sn/NS-CNFs@rGO still interwoven together into 3D nested networks, and the Sn/NS-CNFs are completely sheathed by rGO, even after strong ultrasonic treatment in the preparation process of TEM samples. This demonstrates the tight connection between Sn/NS-CNFs and rGO, which is essential to the structural stability of the final flexible electrode. Furthermore, rGO “bridges” among Sn/NS-CNFs can efficiently add the pathways for charge transfer and contribute extra Na^+^ storage. Meanwhile, the HRTEM image (Fig. 2h) reveals crystalline fringes of 2.91 Å and 3.5 Å can be assigned to the (200) plane of Sn and (002) plane of rGO, respectively, and further confirming that the rGO nanosheets were closely attached to the surface of Sn/NS-CNFs. It is also observed that the Sn/NS-CNFs nanofiber surface is rougher than that of Sn/N-CNFs, which could be expected to provide more active sites in electrochemical reactions. The corresponding EDS elemental mapping from the sample area in the HRTEM image (Fig. 2i) was used to characterize the distribution of elements in Sn/NS-CNFs@rGO. Notably, Sn and S elements were all uniformly distributed along the nanofiber. Moreover, C and N show a very similar distribution, suggesting the N atoms were doped into rGO.

**Fig. 3:**
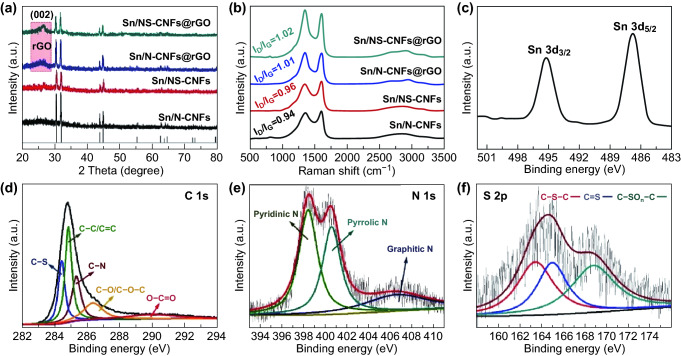
**a** XRD patterns and **b** Raman spectra of Sn/N-CNFs, Sn/NS-CNFs, Sn/N-CNFs@rGO, and Sn/NS-CNFs@rGO. High-resolution XPS spectra of **c** Sn 3*d*, **d** C 1*s*, **e** N 1*s*, and **f** S 2*p*

XRD patterns of Sn/N-CNFs, Sn/NS-CNFs, Sn/N-CNFs@rGO, and Sn/NS-CNFs@rGO samples are exhibited in Fig. 3a. The sharp peaks correspond accurately to metallic Sn (tetragonal phase, JCPDS No. 04-0673), and no impurity peaks belonging to Sn oxides were detected, indicating that Sn^2+^ was completely reduced to metallic Sn after the carbothermal reduction reaction [24, 40]. Notably, comparing with Sn/N-CNFs and Sn/NS-CNFs, both Sn/N-CNFs@rGO and Sn/NS-CNFs@rGO have a broad diffraction peak around 25°, which can be attributed to the (002) plane of graphitic carbon in rGO [41, 42]. The carbon information of Sn/N-CNFs, Sn/NS-CNFs, Sn/N-CNFs@rGO, and Sn/NS-CNFs@rGO were investigated by Raman spectroscopy. As shown in Fig. 3b, three distinct bands at around 1350 (D-band, attributed to disordered turbostratic carbon), 1582, and 2890 cm^−1^ (G-band and D + D’-band, attributed to graphitic carbon) were observed in all samples [43]. The D-band and G-band signals reflect the structure of *sp*^3^ and *sp*^2^ hybridized carbon atoms, and the relative intensity *I*_D_/*I*_G_ is widely taken as a basis to evaluate the graphitization degree and the defect densities of carbonaceous materials [44]. According to the calculations, the *I*_D_/*I*_G_ ratios were 0.94, 0.96, 1.01, and 1.02 for Sn/N-CNFs, Sn/NS-CNFs, Sn/N-CNFs@rGO, and Sn/NS-CNFs@rGO, respectively. The increased ratio of *I*_D_/*I*_G_ suggests that more defects were created after GO reduced and S doping, therefore providing more transport paths for Na^+^ diffusion [27]. Thermogravimetric (TG) analysis was employed to demonstrate the content of Sn in the Sn/N-CNFs and Sn/NS-CNFs@rGO (Fig. S8). The mass contents of Sn in the Sn/N-CNFs and Sn/NS-CNFs@rGO are calculated to be 35.5 and 29.5 wt%, respectively. Accordingly, the content of rGO in the Sn/NS-CNFs@rGO can be calculated to 6 wt%.

The elemental composition and chemical states of Sn/NS-CNFs@rGO were further analyzed by XPS, as shown in Fig. 3c–f. Figure 3c presents two peaks at 485.3 and 495.1 eV, which were attributed to Sn 3*d*_5/2_ and Sn 3*d*_3/2_, respectively, indicating that Sn elements exist mainly in the form of metallic Sn in the Sn/NS-CNFs@rGO. This result agrees well with the XRD and TEM analysis, and consistent with previous observations [20, 42, 45]. The high-resolution XPS spectrum of C 1*s* (Fig. 3d) can be fitted into five predominate peaks at 284.3 (C–S), 284.7 (C–C/C=C), 285.4 (C–N), 286.5 (C–O/C–O–C), and 288.7 (O–C=O) eV, respectively. The N 1*s* spectrum in Fig. 3e contains three main peaks at 398.2, 400.5, and 406.3 eV, which is associated with the pyridinic, pyrrolic, and graphitic types of N atoms, respectively [35, 46]. In the high-resolution S 2*p* spectrum (Fig. 3f), the main peak can also be deconvoluted into three different peaks. The two peaks centered at around 163.5 eV for S 2*p*_3/2_ and 165.0 eV for S 2*p*_1/2_ can be attributed to C–S–C and C=S covalent bonds, respectively. The third peak centered at 168.4 eV can be assigned to the oxidized sulfur species (C–SO_*n*_–C) [35]. These results clearly demonstrate that N and S heteroatoms have been successfully co-doped into the CNFs. It is worth noting that the co-doped N and S atoms could not only change the electron donor properties to increase the charge carrier concentration, but also decorate the carbon matrix with more defects for Na^+^ adsorption.

**Fig. 4:**
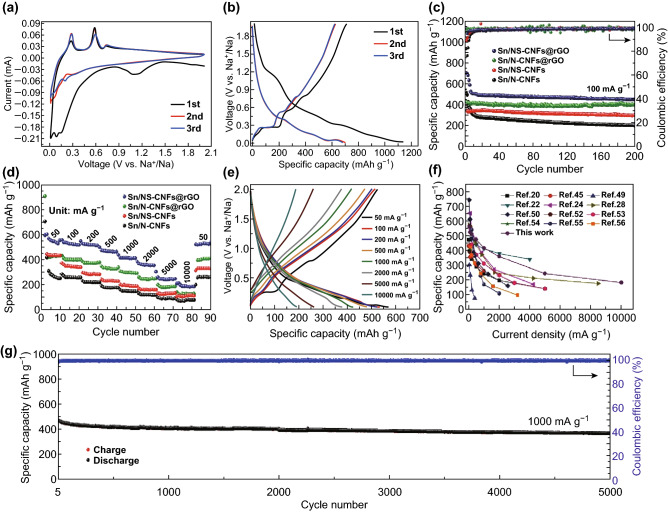
**a** Cyclic voltammograms for the first three cycles of a Sn/NS-CNFs@rGO electrode at a scanning rate of 0.2 mV s^−1^. **b** The first three galvanostatic discharge/charge voltage profiles of a Sn/NS-CNFs@rGO electrode at 100 mA g^−1^. **c** Cycling performance of Sn/N-CNFs, Sn/NS-CNFs, Sn/N-CNFs@rGO, and Sn/NS-CNFs@rGO electrodes at a current density of 100 mA g^−1^ in the voltage range of 0.01–2.0 V vs Na^+^/Na. **d** Rate capabilities of the Sn/N-CNFs, Sn/NS-CNFs, Sn/N-CNFs@rGO, and Sn/NS-CNFs@rGO electrodes at various current densities. **e** Galvanostatic discharge–charge profiles of the Sn/NS-CNFs@rGO at different rates. **f** Comparison of the specific capacities of various Sn-based anode materials at different current densities. **g** Long-term cycling performance of a Sn/NS-CNFs@rGO electrode at 1000 mA g^−1^, and the corresponding Coulombic efficiency

The Sn/NS-CNFs@rGO was directly used for free-standing and flexible electrodes for SIBs, without any binder or conductive additive. Notably, all the electrochemical performances in this work are calculated based on the total mass of Sn/NS-CNFs@rGO composite. The electrochemical reactivity of Sn/NS-CNFs@rGO was characterized by cyclic voltammetry (CV) between 0.01 and 2.0 V, at a scan rate of 0.2 mV s^−1^ (Fig. 4a). During the initial cathodic scan, a broad reduction peak appeared at about 1.1 V and then disappeared in the subsequent scans, which can be denoted as irreversible electrolyte decomposition during the formation of the solid–electrolyte interface (SEI) film. The peaks located at 0.15 and 0.09 V in subsequent cathodic scans are intrinsically associated with the formation of Na_*x*_Sn alloy and the insertion reaction of Na^+^ into carbon. Three oxidation peaks in the anodic scans at 0.28, 0.58, and 0.73 V correspond to desodiation of Na_15_Sn_4_, NaSn, and NaSn_5_, respectively. The desodiation potentials observed in this work are well-agreemented with the experimental and calculated results for Sn-based anodes in the literature [24, 47–49]. The overall reaction process of Sn/NS-CNFs@rGO composite in SIBs is given by Eqs. 1 and 2:

1 $$ 4{\text{Sn}} + 15{\text{Na}}^{ + } \leftrightarrow {\text{Na}}_{15} {\text{Sn}}_{4} $$

2 $$ {\text{xC}} + {\text{Na}}^{ + } + {\text{e}}^{ - } \leftrightarrow {\text{NaC}}_{x} $$

Figure 4b shows the first three Sn/NS-CNFs@rGO electrode galvanostatic discharge/charge voltage profiles at 100 mA g^−1^. The stepwise discharge plateaus appear below 0.5 V corresponding to the sodiation process. In the charge profiles, three obvious potential plateaus are presented at 0.26, 0.55, and 0.77 V, which is the characteristic of stepwise dealloying Na_15_Sn_4_; these results agree well with CV analysis.

The cycling performance of Sn/N-CNFs, Sn/NS-CNFs, Sn/N-CNFs@rGO, and Sn/NS-CNFs@rGO electrodes at a current density of 100 mA g^−1^ between 0.01-2.0 V versus Na^+^/Na are compared in Fig. 4c. Impressively, the Sn/NS-CNFs@rGO exhibits an initial discharge capacity of 1136 mAh g^−1^ and charge capacity of 707 mAh g^−1^, corresponding to the high initial Coulombic efficiency (CE) of 62.2%, suggesting that the encapsulation of Sn QDs in the 3D conductive NS-CNFs@rGO network can effectively alleviate detrimental reactions between Sn and electrolyte. Moreover, the Sn/NS-CNFs@rGO electrodes exhibit an outstanding stability with a discharge capacity of 454 mAh g^−1^ after 200 cycles. The excellent cycling performance can be mainly attributed to the unique structure of Sn/NS-CNFs@rGO. On the one hand, the ultra-small Sn QDs can increase the specific active surface with electrolyte, enhancing the interfacial Na^+^ storage properties and improving capacity [20, 50]. On the other hand, the optimized N,S co-doped CNFs can induce numerous extrinsic defects and active sites, which can further enhance the sodium absorption properties [35]. In comparison, the Sn/N-CNFs, Sn/NS-CNFs, and Sn/N-CNFs@rGO electrodes deliver a relatively lower capacity of 201, 300, and 410 mAh g^−1^ after 200 cycles at 100 mA g^−1^, respectively. It can be concluded that the capacity retention and cycling performance upgrade in the order Sn/N-CNFs < Sn/NS-CNFs < Sn/N-CNFs@rGO < Sn/NS-CNFs@rGO, and also demonstrate that S doping and rGO wrapped can further improve the Na^+^ storage properties [51].

Figure 4d shows the comparison of the rate capabilities of Sn/N-CNFs, Sn/NS-CNFs, Sn/N-CNFs@rGO, and Sn/NS-CNFs@rGO electrodes at different current densities. Clearly, Sn/NS-CNFs@rGO electrode displays superior high rate performance, with high average discharge capacities of 568, 531, 519, 482, 425, 368, and 252 mAh g^−1^ at 50, 100, 200, 500, 1000, 2000, and 5000 mA g^−1^, respectively. Even at a high current density of 10 A g^−1^, the discharge capacity can still reach 189 mAh g^−1^. Remarkably, after undergoing a high discharge/charge rate, the Sn/NS-CNFs@rGO electrode exhibits a high average discharge capacity of 512 mAh g^−1^ when the current density is returned to 50 mA g^−1^, showing a strong tolerance for rapid sodiation and desodiation. All these values outperform the Sn/N-CNFs (285, 260, 222, 182, 177, 130, 96, 80, and 260 mAh g^−1^), Sn/NS-CNFs (426, 351, 294, 233, 228, 168, 131, 105, and 330 mAh g^−1^), and Sn/N-CNFs@rGO (435, 400, 374, 334, 325, 259, 200, 141, and 398 mAh g^−1^), at the current density of 50, 100, 200, 500, 1000, 2000, 5000, and back to 50 mA g^−1^. Such an excellent rate performance of Sn/NS-CNFs@rGO can be ascribed to the fast ion transport and enhanced electrical conductivity, profiting from the 3D hierarchical conductive network of NS-CNFs and rGO. The corresponding charge/discharge profiles of Sn/NS-CNFs@rGO at different rates are exhibited in Fig. 4e, further demonstrating the high-rate capability and high-capacity retention. It is noteworthy that the 3D flexible conductive Sn/NS-CNFs@rGO network fabricated in this work exhibited excellent high-rate capability and outstanding cycling stability as compared with various previously reported Sn-based anode materials (Fig. 4f) [20, 22, 24, 28, 45, 49, 50, 52–56] and other reported 3D free-standing electrodes (Table S1). For comparison, Sn/NS-CNFs@rGO electrodes with lower/higher Sn content (denoted as L–Sn/NS-CNFs@rGO and H–Sn/NS-CNFs@rGO) were also prepared by altering the mass ratio of metal salts and carbon source, where the Sn content of L–Sn/NS-CNFs@rGO and H–Sn/NS-CNFs@rGO is determined to be ~ 23.2 wt% and ~ 37.4 wt% from TG analysis (Fig. S9). As displayed in Fig. S10, L–Sn/NS-CNFs@rGO displays much lower specific capacity due to its limited Sn content; while H–Sn/NS-CNFs@rGO electrode delivers a relatively high initial reversible capacity of 598 mAh g^−1^ at 50 mA g^−1^, but displays inferior rate capability (only 130 mAh g^−1^ at 10 A g^−1^). The higher Sn QDs content of H–Sn/NS-CNFs@rGO appeared to not endure the generated mechanical stress during cycling, and pulverized to some extent with aggregation, resulting in a deterioration of cyclability.

In addition, Sn/NS-CNFs@rGO electrodes were conducted at high current densities to further demonstrate the long-term cycling stability. In order to fully evaluate the electrode, the Sn/NS-CNFs@rGO was activated for five cycles at a relatively lower current density of 100 mA g^−1^. As shown in Fig. 4g, even after 5000 cycles at a current density of 1 A g^−1^, Sn/NS-CNFs@rGO electrode exhibits a high reversible specific capacity of 373 mAh g^−1^, corresponding to the energy density of 210 Wh kg^−1^ and power density of 572 W kg^−1^ (based on the total mass of electrode). Remarkably, the corresponding average CE is up to 99.93%. More importantly, reversible specific capacities of 343 and 220 mAh g^−1^ were obtained after 1000 cycles at 3 A g^−1^ and after 2000 cycles at 5 A g^−1^, respectively (Fig. S11), further demonstrating the superior cycling stability at high rates. This outstanding cycling performance of Sn/NS-CNFs@rGO electrode should be related to the synergetic effect of its smart 3D hierarchical conductive network, which is beneficial to enhance the electron and sodium-ion transport kinetics, the external elastic rGO protection, and the mitigated volume expansion. Electrochemical impedance spectroscopy (EIS) measurements were carried out to investigate the charge transport kinetics of various electrodes after five full discharge/charge cycles, as exhibited in Fig. S12. It is notable that the radius of the semicircle for Sn/NS-CNFs@rGO is much smaller than that for Sn/N-CNFs, Sn/NS-CNFs, and Sn/N-CNFs@rGO. The Na^+^ diffusion coefficient of Sn/NS-CNFs@rGO is also calculated to be the largest (Fig. S13, Eqs. S1 and S2), demonstrating that Sn/NS-CNFs@rGO shows the highest electrochemical activity for sodium storage [27, 57].

**Fig. 5:**
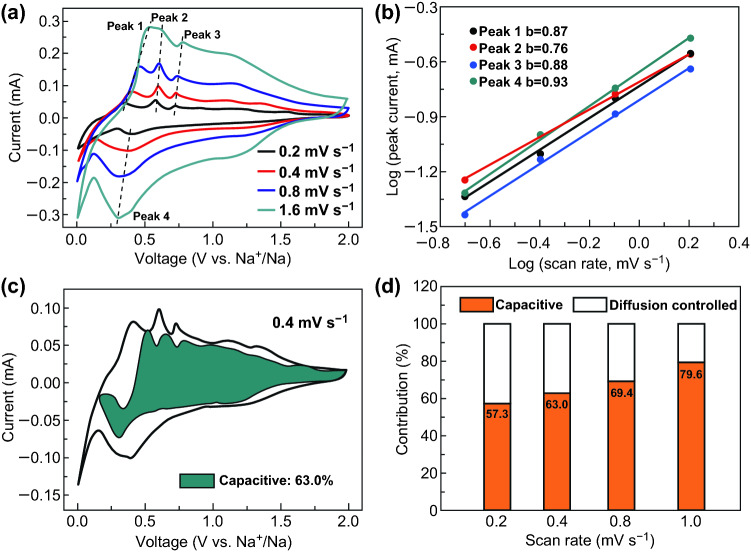
**a** CV profiles of Sn/NS-CNFs@rGO at various scan rate. **b** Plots of (log *i*) versus (log *υ*). **c** Capacitive contribution in the CV curve (shaded region) of Sn/NS-CNFs@rGO obtained at 4 mV s^−1^. **d** Capacitive contribution in the CV profiles obtained at various scan rates

In order to better understanding the excellent electrochemical properties of Sn/NS-CNFs@rGO electrode, CV test at various scanning rates was conducted to assess the Na^+^ storage mechanism, as shown in Fig. 5a. The relationship between scan rate (*υ*) and peak current (*i*) can be used to distinguish the capacity contribution basis of Eq. 3:

3 $$ i = \alpha \upsilon^{b} $$

where *α* and *b* are constants, and the value of *b* can be determined by the slope from plot of (log *i*) vs (log *υ*). Particularly, *b *= 0.5 reveals a diffusion-controlled process, while *b *= 1.0 implies a capacitive-controlled process. As shown in Fig. 5b, the calculated *b* values for the cathodic and anodic peaks are all close to 1.0, indicating the substantial capacitive-controlled contribution. In addition, the two processes can be estimated from Eq. 4:

4 $$ I(\upsilon ) = k_{1} \upsilon + k_{2} \upsilon^{1/2} $$

where *i* is the total current at a fixed potential, *k*_*1*_*υ* and *k*_*2*_*υ*^*1/2*^ represent the contributions of surface capacitive effects and diffusion-controlled process. In Fig. 5c, the capacitive process contributes 63.0% of total sodium-ion storage at 0.4 mV s^−1^. Moreover, the ratio gradually improves with the increasing scan rates (Fig. 5d), reaching 79.6% at the scan rate of 1.6 mV mV s^−1^.

**Fig. 6:**
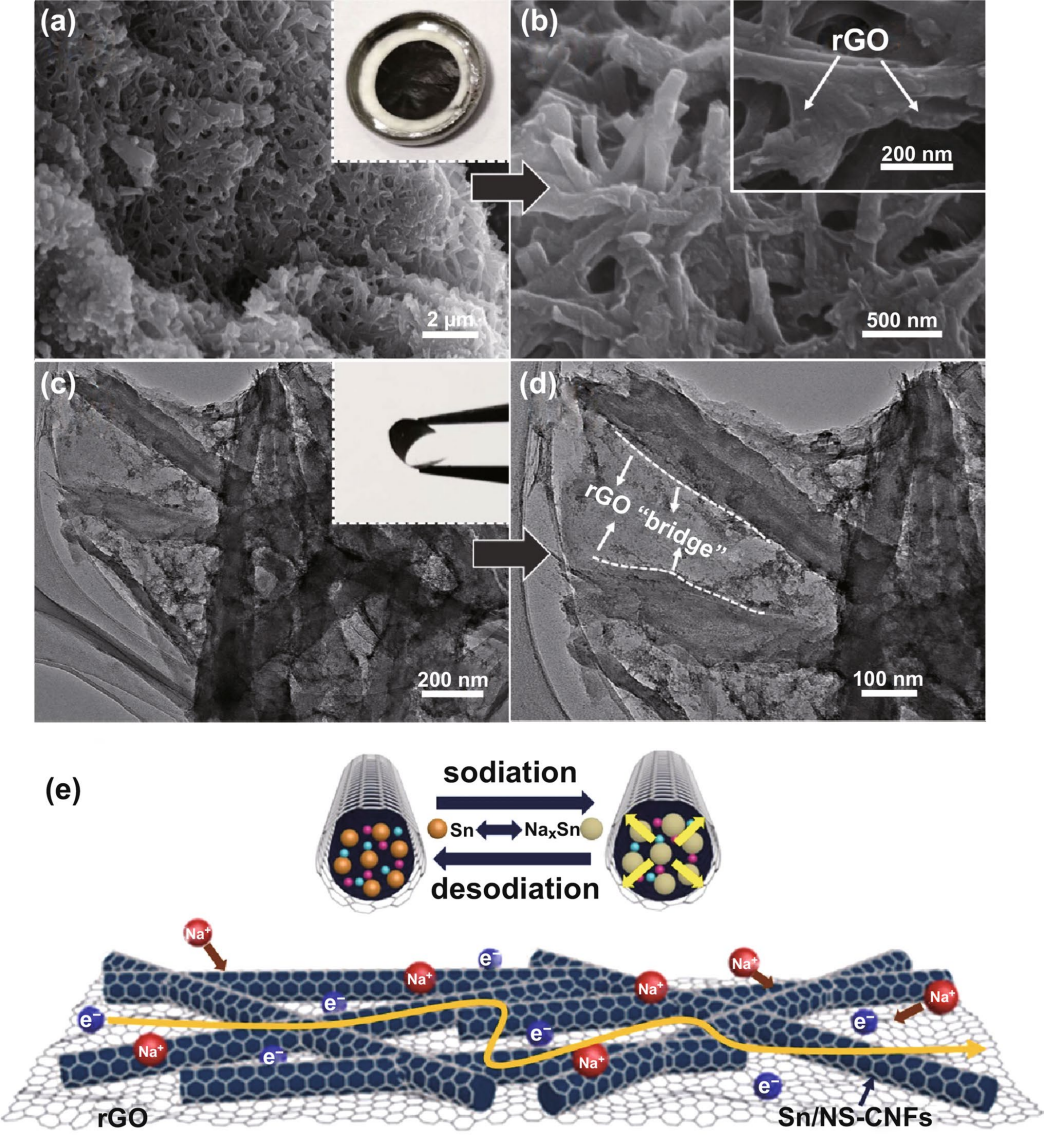
**a**, **b** *Ex situ* SEM and **c**, **d** ex situ TEM images of a Sn/NS-CNFs@rGO electrode after discharging/charging at 3 A g^−1^ for 1000 cycles, and the insets are digital photos of flexible Sn/NS-CNFs@rGO electrodes after discharging/charging at 3 A g^−1^ for 1000 cycles. **e** Schematic illustration of the enhanced sodium-ion and electron transportation mechanisms of Sn/NS-CNFs@rGO

To investigate the structural stability after high-rate cycling tests, the morphology of Sn/NS-CNFs@rGO electrodes after discharging/charging at 3 A g^−1^ for 1000 cycles was observed using ex situ SEM and ex situ TEM analysis. The insets in Fig. 6a, c presents a free-standing and flexible electrode after cycling without any mechanical cracks, which further proves the resilience of Sn/NS-CNFs@rGO. As revealed in Fig. 6a, b, after undergoing high-rate tests, the Sn/NS-CNFs@rGO still retains its original structure, and the sheathing rGO can be clearly distinguished as before. Figure 6c, d shows Sn/NS-CNFs maintained their 1D structure, and a multiplicity of rGO “bridges” is generated by the rGO tightly wrapping on the CNF surface, indicating the consistent structural connections of the Sn/NS-CNFs@rGO. Meanwhile, Sn QDs keep remained evenly dispersed in NS-CNFs without evident aggregation, demonstrating that the NS-CNFs can act as a good buffer material to prevent aggregation of Sn QDs.

Based on the above results, the excellent cycle stability and ultra-high-rate capability of Sn/NS-CNFs@rGO architecture may be attributed to the following mechanisms, as shown in Fig. 6e. Firstly, the ultra-small Sn QDs can significantly enhance Na^+^ insertion by reducing diffusion/migration barrier, and thus enhance the utilization rate of active materials. Secondly, the rGO wrapping around the Sn/NS-CNFs serves multiple functions: (1) with large 2D surface and superior electrical conductivity, rGO could improve the conductivity of the electrode and enlarge the contact area with electrolyte; (2) rGO possesses excellent mechanical flexibility and chemical stability, which could efficiently alleviate the huge volume expansion/contraction of the electrode during sodiation/desodiation processes, maintaining the electrode integrity; (3) the rGO “bridges” among Sn/NS-CNFs function as an electrical highway and also contribute extra sodium storage. Thirdly, the N,S co-doped CNFs can induce numerous extrinsic defects and active sites, which can further enhance the sodium absorption properties. Meanwhile, the 1D N,S co-doped CNFs and 2D rGO can interlink into a 3D hierarchical conductive network, which could not only further promote the Na^+^ and electrons diffusion kinetics, but also accommodates the volume expansion of Sn QDs and prevents both pulverization and aggregation.

## Conclusions

In summary, using a facile electrospinning technique followed by filtration and calcination treatment, we successfully constructed a 3D hierarchically conductive Sn/NS-CNFs@rGO network, which can be directly employed as flexible and free-standing electrodes in SIBs. Benefiting from the fast electron/ion transfer and synergistic effects of 3D conductive network composite of ultra-long 1D NS-CNFs and 2D rGO, the as-prepared electrode exhibits a superior electrochemical performance, including ultra-long cycling performance (373 mAh g^−1^ after 5000 cycles at a current density of 1 A g^−1^, corresponding to the energy density of 210 Wh kg^−1^ and power density of 572 W kg^−1^), and high-rate capacity (189 mAh g^−1^ at a current density of 10 A g^−1^). More importantly, this 3D hierarchical structural engineering method developed in this work may provide a facile and efficient strategy for the construction of flexible electrodes for other energy storage devices.

## Electronic supplementary material

Below is the link to the electronic supplementary material.

Supplementary material 1 (PDF 1029 kb)
