# Supplementary material for: Flexible Conductive Anodes Based on 3D Hierarchical Sn/NS-CNFs@rGO Network for Sodium-Ion Batteries
Source: Nanomicro Lett. 2019 Aug 1;11:63. doi: 10.1007/s40820-019-0294-9 (PMC7770738; doi:10.1007/s40820-019-0294-9)
Supplement: Supplementary file 1 — Supplementary material 1 (PDF 1029 kb) [file 40820_2019_294_MOESM1_ESM.pdf]

Supporting Information for

Flexible Conductive Anodes Based on 3D Hierarchical Sn/NS CNFs@rGO Network for Sodium-Ion Batteries

Linqu Luo[1], Jianjun Song[1, *], Longfei Song[1], Hongchao Zhang[1], Yicheng Bi[2], Lei Liu[3], Longwei Yin[4], Fengyun Wang[1, *], Guoxiu Wang[5, *]

[1]College of Physics and State Key Laboratory of Bio-Fibers and Eco-Textiles, Qingdao University, Qingdao 266071, People's Republic of China

[2]College of Electromechanical Engineering, Qingdao University of Science & Technology, No.99 Songling Road, Qingdao, Shandong 260061, People's Republic of China

[3]School of Materials Science and Engineering, Shandong University of Science and Technology, Qingdao 266590, People's Republic of China

[4]School of Materials Science and Engineering, Shandong University, Jinan 250061, People's Republic of China

[5]Centre for Clean Energy Technology, University of Technology Sydney, Broadway, Sydney, NSW 2007, Australia

*Corresponding authors. E-mail: jianjun.song@qdu.edu.cn (Jianjun Song); fywang@qdu.edu.cn (Fengyun Wang); Guoxiu.Wang@uts.edu.au (Guoxiu Wang)

Supplementary Figures and Table

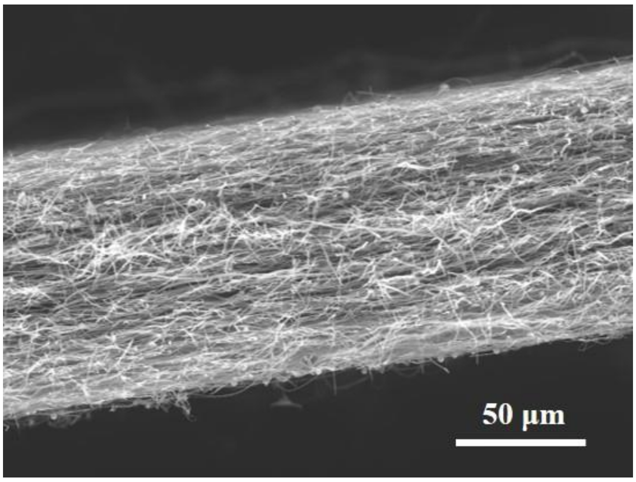

Fig. S1 Cross-sectional SEM image of the Sn/N-CNFs membrane with thickness of 109 µm

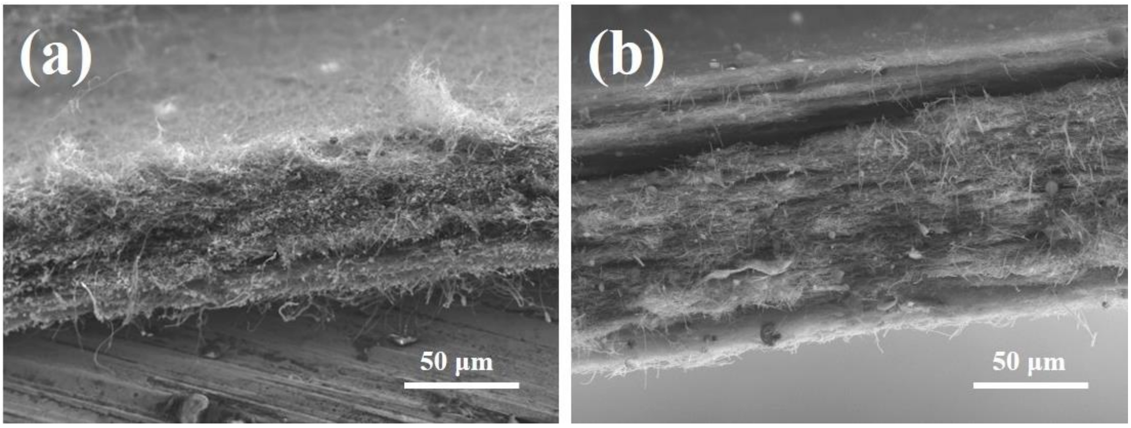

Fig. S2 Cross-sectional SEM image of the Sn/N-CNFs membrane with different thickness of **a** 78 μm, **b** 139 μm

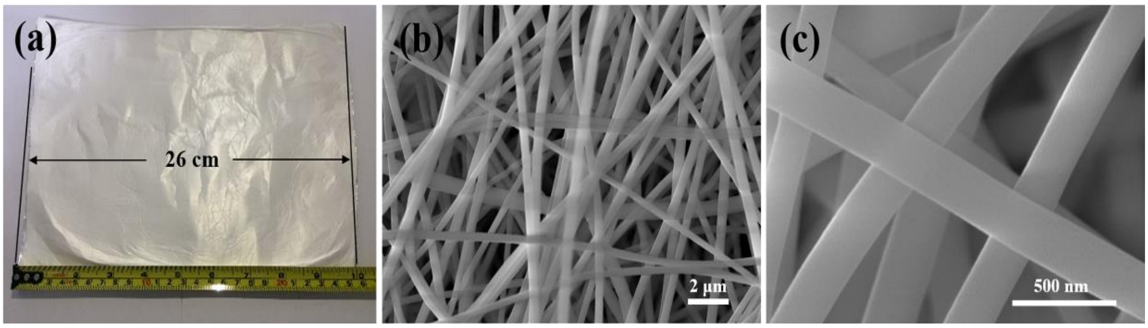

Fig. S3 a Digital photos of as-spun PAN/$SnCl_2$/thiourea nanofibers membrane; **b** and **c** SEM images of PAN/$SnCl_2$/thiourea precursor nanofibers

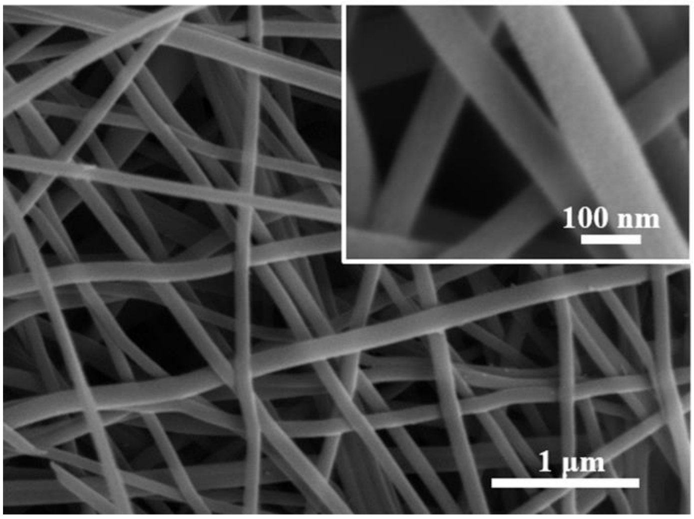

Fig. S4 SEM images of Sn/NS-CNFs

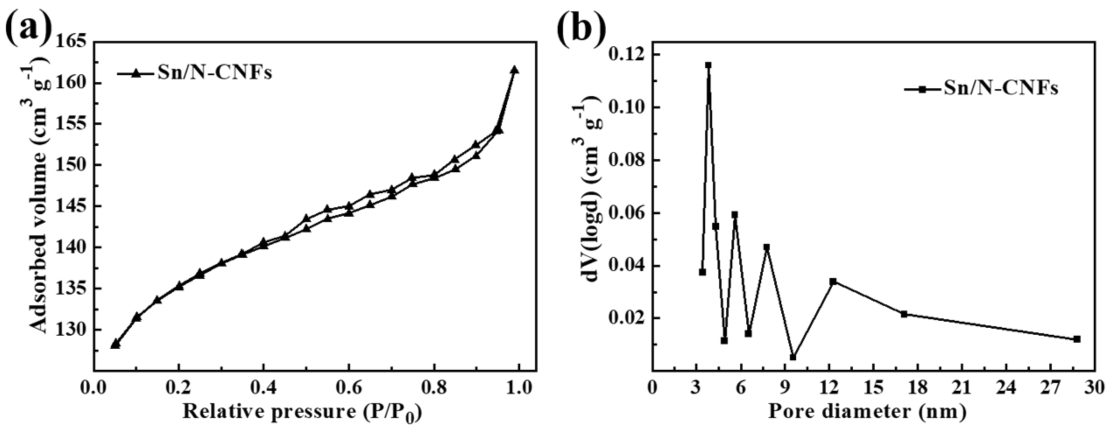

Fig. S5 a N_2 adsorption–desorption isotherms and **b** the corresponding pore size distribution curves of Sn/N-CNFs

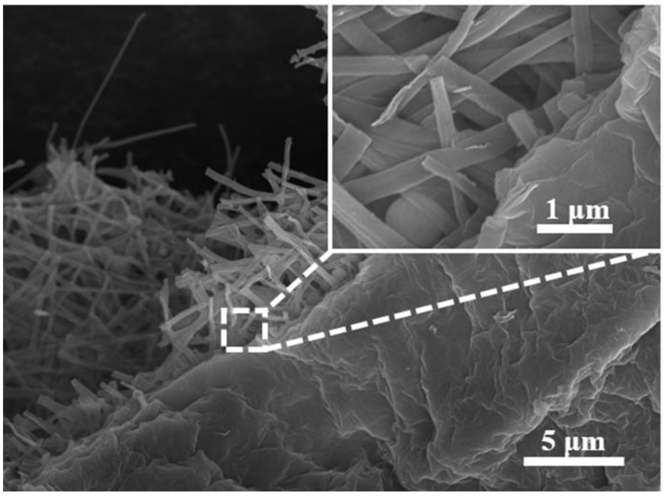

Fig. S6 SEM images of Sn/N-CNFs@rGO

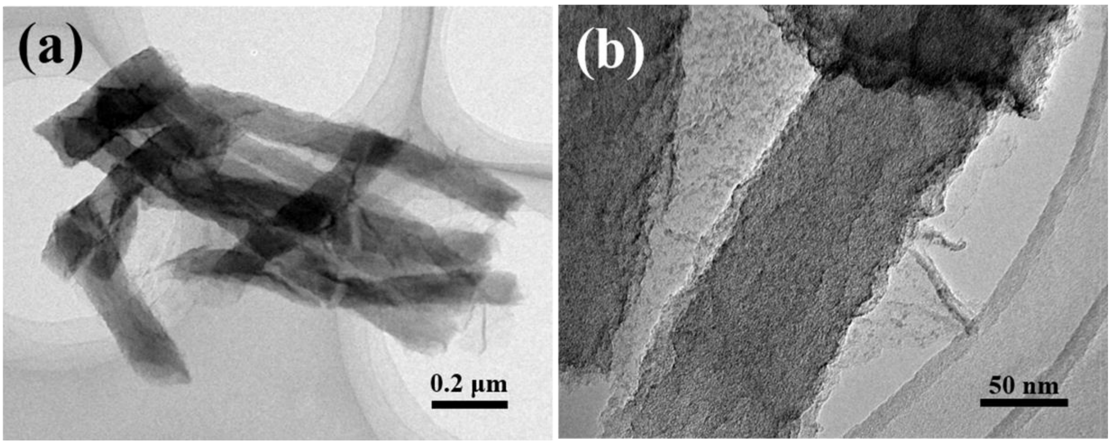

Fig. S7 a Low- and **b** high-magnification TEM images of Sn/NS-CNFs@rGO

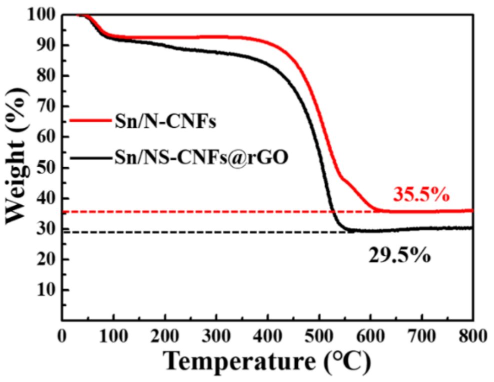

Fig. S8 The TGA data of Sn/N-CNFs and Sn/NS-CNFs@rGO

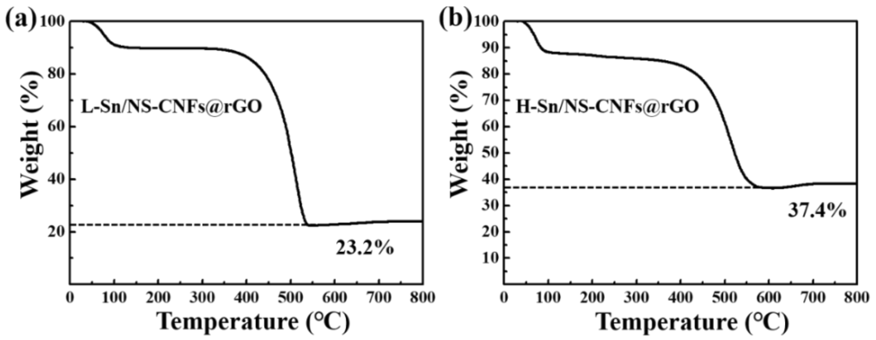

Fig. S9 a The TGA data of Sn/NS-CNFs@rGO with low Sn content (L-Sn/NS-CNFs@rGO), and **b** high Sn content (H-Sn/NS-CNFs@rGO)

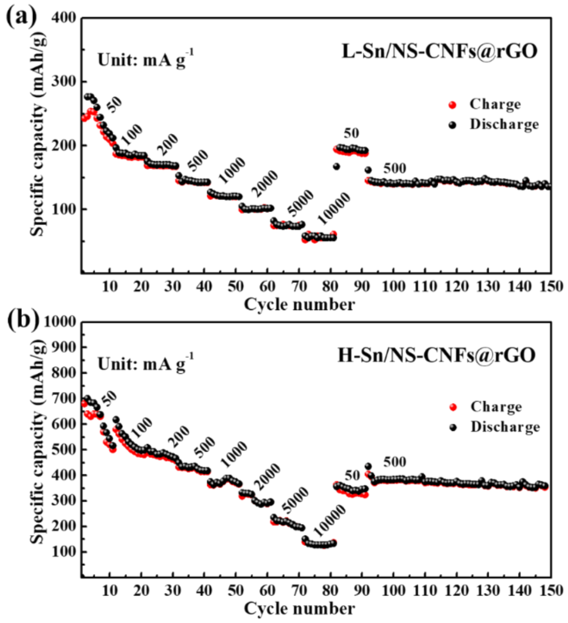

Fig. S10 a Rate capability and cycling performance of L-Sn/NS-CNFs@rGO electrode, and **b** H-Sn/NS-CNFs@rGO electrode

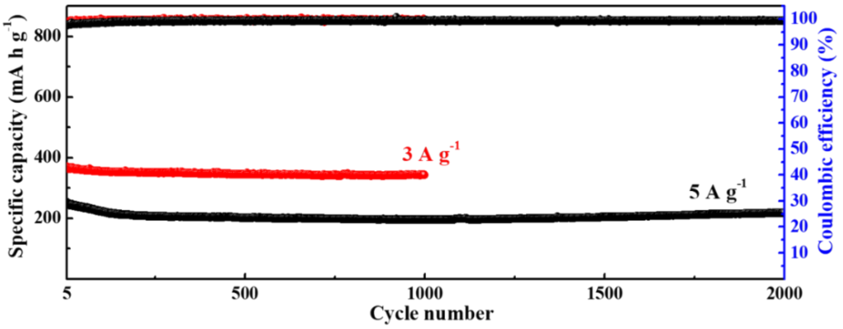

Fig. S11 Long-term cycling performance of the Sn/NS-CNFs@rGO electrodes at 3 A g^{-1} and 5 A g^{-1}, and the corresponding coulombic efficiency

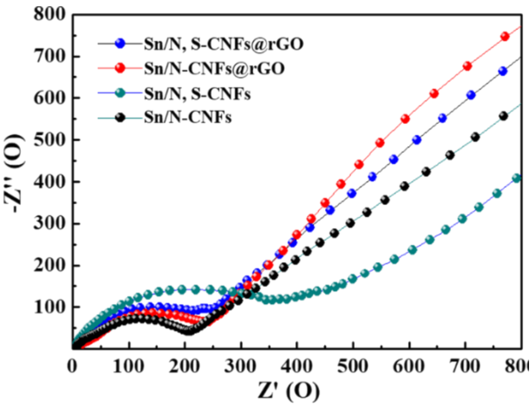

Fig. S12 EIS curves of Sn/N-CNFs, Sn/NS-CNFs, Sn/N-CNFs@rGO, and Sn/NS-CNFs@rGO electrodes after 5 cycles

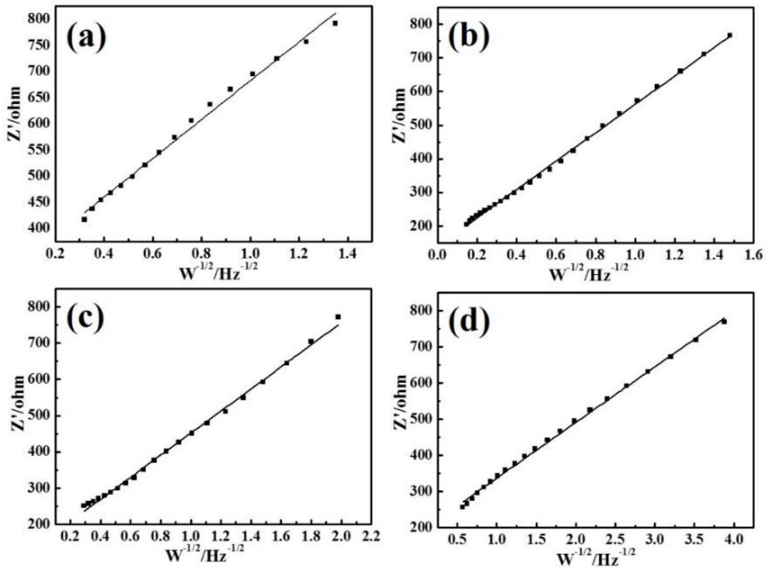

Fig. S13 Real parts of the impedance (Z’) versus the reciprocal square root of angular frequency (ω) in the low frequency region of **a** Sn/N-CNFs, **b** Sn/NS-CNFs, **c** Sn/N-CNFs@rGO, and **d** Sn/NS-CNFs@rGO

The sodium ion diffusion coefficients (D_{Na}) is an important parameter of kinetics for an electrochemical reaction. It is calculated using Eq. S1:

$$D_{Na} = \frac{R^2T^2}{2A^2n^4F^4C^2\sigma^2} \qquad \text{(S1)}$$

where R is the gas constant, T is the absolute temperature, A is the surface area of the electrode, n is the number of electrons per molecule during oxidization, F is the Faraday constant, C is the concentration of sodium ion, and σ is the Warburg factor which has relationship with Z' :

$$Z' = R_D + R_C + \sigma\omega^{-\frac{1}{2}} \qquad \text{(S2)}$$

Figure S13 shows the relationship between Z' and square root of frequency ($\omega^{-1/2}$) in the low-frequency region. The diffusion coefficient of sodium ion is calculated based on Eqs. S1 and S2. The calculated sodium ion diffusion coefficients of Sn/N-CNFs, Sn/NS-CNFs, Sn/N-CNFs@rGO, and Sn/NS-CNFs@rGO are 2.72×10^{-13}, 2.08×10^{-13}, 3.99×10^{-13}, 1.58×10^{-12} $cm^2\ s^{-1}$, respectively. Obviously, the sodium ion diffusion ability of Sn/NS-CNFs@rGO is greatly enhanced compared with Sn/N-CNFs, Sn/NS-CNFs, Sn/N-CNFs@rGO.

Table S1 Comparisons of the sodium storage properties for previously reported 3D free-standing electrodes

Structures	Electrochemical performance	Refs.
MoS_2/Graphene	230 h g^{-1} after 20 cycles at 25 mA g^{-1}	[S1]
Nitrogen-Doped Carbon Sheets	76 mAh g^{-1} after 2000 cycles at 4.5 C	[S2]
CC@CN@MoS_2	265 mAh g^{-1} after 1000 cycles at 1 A g^{-1}	[S3]
FeS@C/carbon cloth	365 mAh g^{-1} after 100 cycles at 0.15 C	[S4]
TiO_2-Sn@CNFs	413 mAh g^{-1} after 400 cycles at 100 mA g^{-1}	[S5]
Hydrogen substituted graphdiyne	360 mAh g^{-1} after 1000 cycles at 1 A g^{-1}	[S6]
ReS_2/N-CNFs	245 mAh g^{-1} after 800 cycles at 100 mA g^{-1}	[S7]
Fe_3O_4@MoS_2-GP	388 mAh g^{-1} after 300 cycles at 100 mA g^{-1}	[S8]
MoO_{3-x} grown on flexible carbon cloth	156 mAh g^{-1} after 200 cycles at 100 mA g^{-1}	[S9]
SnS_2–RGONRP	334 mAh g^{-1} after 1500 cycles at 1 A g^{-1}	[S10]
Free-standing fluorine and nitrogen co-doped graphene paper	203 mAh g^{-1} after 100 cycles at 50 mA g^{-1}	[S11]
NCF@rGO-TiO_2	214 mAh g^{-1} after 150 cycles at 1C	[S12]
$CoSe_2$/CNFs	430 mAh g^{-1} after 400 cycles at 200 mA g^{-1}	[S13]
MoS_2-F	243 mAh g^{-1} after 1100 cycles at 1 A g^{-1}	[S14]
SnS/C NFs	481 mAh g^{-1} after 100 cycles at 50 mA g^{-1}; 349 mAh g^{-1} after 500 cycles at 200 mA g^{-1}	[S15]
Sn/NS-CNFs@rGO	454 mAh g^{-1} after 200 cycles at 100 mA g^{-1}; 373 mAh g^{-1} after 5000 cycles at 1 A g^{-1}; 189 mA h g^{-1} at 10 A g^{-1}	**This work**

Supplementary References

[S1] L. David, R. Bhandavat, G. Singh, MoS_2/graphene composite paper for sodium-ion battery electrodes. ACS Nano **8**(2), 1759-1770 (2014). https://doi.org/10.1021/nn406156b

[S2] T.Z. Yang, T. Qian, M.F. Wang, X.W. Shen, N. Xu, Z.Z. Sun, C.L. Yan, A sustainable route from biomass byproduct okara to high content nitrogen-doped carbon sheets for efficient sodium ion batteries. Adv. Mater. **28**(3), 539-545 (2016). https://doi.org/10.1002/adma.201503221

[S3] W.N. Ren, H.F. Zhang, C. Guan, C.W. Cheng, Ultrathin MoS_2 nanosheets@metal organic framework-derived n-doped carbon nanowall arrays as sodium ion battery anode with superior cycling life and rate capability. Adv. Funct. Mater. **27**(32), 1702116 (2017). https://doi.org/10.1002/adfm.201702116

[S4] X. Wei, W.H. Li, J.A. Shi, L. Gu, Y. Yu, FeS@C on carbon cloth as flexible electrode for both lithium and sodium storage. ACS Appl. Mater. Interfaces **7**(50), 27804-27809 (2015). https://doi.org/10.1021/acsami.5b09062

[S5] M. Mao, F. Yan, C. Cui, J. Ma, M. Zhang, T. Wang, C. Wang, Pipe-wire TiO_2-Sn@carbon nanofibers paper anodes for lithium and sodium ion batteries. Nano Lett. **17**(6), 3830 (2017). https://doi.org/ 10.1021/acs.nanolett.7b01152

[S6] J.J. He, N. Wang, Z.L. Cui, H.P. Du, L. Fu et al., Hydrogen substituted graphdiyne as carbon-rich flexible electrode for lithium and sodium ion batteries. Nat. Commun. **8,** 1172 (2017). https://doi.org/10.1038/s41467-017-01202-2

[S7] M.L. Mao, C.Y. Cui, M.G. Wu, M. Zhang, T. Gao et al., Flexible ReS_2 nanosheets/n-doped carbon nanofibers-based paper as a universal anode for alkali (Li, Na, K) ion battery. Nano Energy **45**, 346-352 (2018). https://doi.org/10.1016/j.nanoen.2018.01.001

[S8] D.Z. Kong, C.W. Cheng, Y. Wang, Z.X. Huang, B. Liu, Y. Von Lim, Q. Ge, H.Y. Yang, Fe_3O_4 quantum dot decorated MoS_2 nanosheet arrays on graphite paper as free-standing sodium-ion battery anodes. J. Mater. Chem. A **5**(19), 9122-9131 (2017). https://doi.org/10.1039/c7ta01172e

[S9] Y.F. Li, D.D. Wang, Q.Y. An, B. Ren, Y.G. Rong, Y. Yao, Flexible electrode for long-life rechargeable sodium-ion batteries: Effect of oxygen vacancy in MoO_{3-x}. J. Mater. Chem. A **4**(15), 5402-5405 (2016). https://doi.org/10.1039/c6ta01342b

[S10] Y. Liu, Y. Yang, X. Wang, Y. Dong, Y. Tang, Z. Yu, Z. Zhao, J. Qiu, Flexible paper-like free-standing electrodes by anchoring ultrafine SnS_2 nanocrystals on graphene nanoribbons for high-performance sodium ion batteries. ACS Appl. Mater. Interfaces **9**(18), 15484 (2017). https://doi.org/ 10.1021/acsami.7b02394

[S11] H. An, L. Yu, G. Yi, C. Chen, J. Han, Y. Feng, F. Wei, Free-standing fluorine and nitrogen co-doped graphene paper as a high-performance electrode for flexible sodium-ion batteries. Carbon **116**, 338-346 (2017). https://doi.org/10.1016/j.carbon.2017.01.101

[S12] X.M. Zhang, B.Y. Wang, W. Yuan, J.H. Wu, H. Liu, H. Wu, Y. Zhang, Reduced graphene oxide modified n-doped carbon foam supporting TiO_2 nanoparticles as flexible electrode for high-performance Li/Na ion batteries. Electrochim. Acta **311**, 141-149 (2019). https://doi.org/10.1016/j.electacta.2019.04.136

[S13] H. Yin, H.Q. Qu, Z.T. Liu, R.Z. Jiang, C. Li, M.Q. Zhu, Long cycle life and high rate capability of three dimensional $CoSe_2$ grain-attached carbon nanofibers for flexible sodium-ion batteries. Nano Energy **58**, 715-723 (2019). https://doi.org/10.1016/j.nanoen.2019.01.062

[S14] Q. Ni, Y. Bai, S.N. Guo, H.X. Ren, G.H. Chen, Z.H. Wang, F. Wu, C. Wu, Carbon nanofiber elastically confined nanoflowers: A highly efficient design for molybdenum disulfide-based flexible anodes toward fast sodium storage. ACS Appl. Mater. Interfaces **11**(5), 5183-5192 (2019). https://doi.org/10.1021/acsami.8b21729

[S15] J. Xia, L. Liu, S. Jamil, J.J. Xie, H.X. Yan et al., Free-standing SnS/C nanofiber anodes for ultralong cycle-life lithium-ion batteries and sodium-ion batteries. Energy Storage Mater. **17**, 1-11 (2019). https://doi.org/10.1016/j.ensm.2018.08.005
